# Acute Exposure to a Precursor of Advanced Glycation End Products Induces a Dual Effect on the Rat Pancreatic Islet Function

**DOI:** 10.1155/2014/378284

**Published:** 2014-11-17

**Authors:** Ghada Elmhiri, Luiz Felipe Barella, Didier Vieau, Sylvaine Camous, Paulo C. F. Mathias, Latifa Abdennebi-Najar

**Affiliations:** ^1^Institut Polytechnique LaSalle Beauvais, EGEAL-UP 2012.10.101., 19 rue Pierre Waguet, 60026 Beauvais Cedex, France; ^2^Laboratory of Secretion Cell Biology, Department of Biotechnology, Genetics and Cell Biology, State University of Maringá, 87020-900 Maringá, PR, Brazil; ^3^Environnement Périnatal et Croissance (EA4489), Equipe Dénutritions Maternelles Périnatales, SN4, Université de Lille 1, 59655 Villeneuve d'Ascq, France; ^4^INRA, UMR1198, Biologie du Développement et Reproduction, 78352 Jouy en Josas, France

## Abstract

*Aim.* Chronic diseases are the leading cause of death worldwide. Advanced glycation end products, known as AGEs, are a major risk factor for diabetes onset and maintenance. Methylglyoxal (MG), a highly reactive metabolite of glucose, is a precursor for the generation of endogenous AGEs. *Methods.* In this current study we incubated *in vitro* pancreatic islets from adult rats in absence or presence of MG (10 *μ*mol/l) with different concentrations of glucose and different metabolic components (acetylcholine, epinephrine, potassium, forskolin, and leucine). *Results.* Different effects of MG on insulin secretion were evidenced. In basal glucose stimulation (5.6 mM), MG induced a significant (*P* < 0.05) increase of insulin secretion. By contrast, in higher glucose concentrations (8.3 mM and 16.7 mM), MG significantly inhibited insulin secretion (*P* < 0.05). In the presence of potassium, forskolin, and epinephrine, MG enhanced insulin secretion (*P* < 0.05), while when it was incubated with acetylcholine and leucine, MG resulted in a decrease of insulin secretion (*P* < 0.05). *Conclusion.* We suggest that MG modulates the secretion activity of beta-cell depending on its level of stimulation by other metabolic factors. These results provide insights on a dual acute effect of MG on the pancreatic cells.

## 1. Introduction

Glycemia and diabetes are rising globally accounting 347 million people worldwide. The study of type 2 diabetes (T2D) has been stimulated due to its growing pandemic and intricate physiopathology, in search of better ways of prevention and treatment of the T2D and its associated complications [1]. Among the several studies to explain the different causative factors and their roles in the onset and maintenance of the T2D and pancreatic islet dysfunctions, the formation of the advanced glycation end products (AGEs) has gained significant importance [2]. For example, it has been reported that patients with diabetes present increased serum concentrations of AGEs compared with nondiabetic patients [3].

AGEs are formed and accumulated by endogenous and exogenous ways. Among the mechanisms, these molecules can increase their intracellular concentrations through the autooxidation of glucose resulting in glyoxal [4] that can undergo a final fragmentation producing the methylglyoxal [5]. Glyoxal and methylglyoxal interact with amino groups of intracellular and extracellular proteins to produce the AGEs. In addition to the prevailing formation of AGEs under physiological conditions and their increase under conditions of hyperglycemia and oxidative stress [6], these products are also introduced in the organism by means of exogenous sources, such as tobacco and diet. The diet is considered the main exogenous source of AGEs and is associated with the development of several pathologies such as diabetes [7]. Food preparation methods using high temperatures (frying, roasting, and grilling) potentialize the production of AGEs present in the diets; moreover, high-fat foods are the main contributors to the formation of AGEs in the diets [8]. Thus, both the endogenous and exogenous AGEs influence the installation and progression of pathologies like T2D.

The pancreatic islet insulin secretion is highly regulated by glucose and secretagogues. Glucose is responsible for the triggering of insulin release while the secretagogues may amplify or inhibit the release depending on their interactions. The triggering and amplifying pathways were widely reviewed in the literature [9–12]. Recent studies have shown that AGEs long-term exposure may impair the beta-cell function by inducing apoptosis or decreasing the insulin secretion [13, 14]. However, the specific sites of interactions of the AGEs on the different pathways of insulin secretion remain to be elucidated [15].

Our current study highlights the acute effect of incubation of a precursor of AGEs, methylglyoxal (MG), on the insulin secretion triggered by glucose and modulated by different secretagogues. Our findings revealed that MG exerts a dual role on the insulin secretion depending on both glucose concentration and the type of secretagogue.

## 2. Materials and Methods

### 2.1. Animals

The experiments were conducted in accordance with the European Communities Council Directive of 1986 (86/609/EEC) and approved by the French Departmental Direction of Veterinary Services Committee.

Ten adult Wistar rats were purchased from Harlan Laboratories (Gannat, France) and housed two per cage. The animal facility rooms were maintained on a dark/light schedule (12 h/12 h, light on at 08 h) and controlled temperature (22 ± 2°C). Animals were maintained with free access to food (regular rat chow diet) and tap water. After a week of acclimation, the rats weighing around 350 grams were used to the subsequent experiments.

### 2.2. Isolation of the Pancreatic Islets

Pancreatic islets were isolated by the collagenase method as previously described [16], with adaptations. The animals were decapitated and the abdominal wall was cut open. Following, 8 mL of Hanks buffered saline solution (HBSS; (mM): NaCl, 136.9; KCl, 5.4; MgSO_4_7H_2_O, 0.81; Na_2_HPO_4_, 0.34; KH_2_PO_4_, 0.44; CaCl_2_2H_2_O, 1.26; NaHCO_3_, 4.16; glucose, 0.06; BSA, 15); and ((v/v; 95% O_2_ + 5% CO_2_, mixed)/10 min, pH 7.4) containing ((w/v) 0.1% collagenase type XI, 5% BSA and 0.6% N-(2-hydroxyethylpiperazine)-N′-(2-ethanesulfonic acid; HEPES); Sigma-Aldrich) was injected into the rats' common bile duct. The inflated pancreas with collagenase was incubated at 37°C. The pancreas was washed with HBSS and the islets were hand-picked with the aid of a stereomicroscope.

### 2.3. Functional Study of the Insulin Secretion

To adapt the pancreatic islets, they were preincubated for 60 min in 1 mL of normal Krebs-Ringer solution ((mM): NaCl, 115; NaHCO_3_, 24; KCl, 1.6; MgCl_6_H_2_O, 1; CaCl_2_2H_2_O, 1; BSA, 15; pH 7.4) containing 5.6 mmol/L glucose. This solution was gassed with (v/v) 95% O_2_ C5% CO_2_ (mixed) to maintain pH 7.4. After the preincubation, groups of 4 islets were incubated with different concentrations of glucose (5.6, 8.3, and 16.7 mM) for an additional 60 min.

To study the different amplifying/inhibiting pathways of insulin secretion, islets were incubated (after the preincubation period) for further 60 min with 8.3 mM of glucose plus 10 *μ*M of acetylcholine in the presence of 10 *μ*M of neostigmine to avoid the acetylcholinesterase action; or 16.7 mM of glucose plus 1 *μ*M of epinephrine; or 5.6 mM of glucose plus 40 mM of K^+^; or 8.3 mM of glucose plus 10 *μ*M of forskolin; or 5.6 mM of glucose plus 10 mM of leucine. All the incubations were also tested in presence of 10 *μ*M of methylglyoxal. Doses were tested before or chosen from the literature to the optimal induction/inhibition of the insulin secretion.

The supernatants from the incubations were collected and stored for posterior insulin measurements using a radioimmunoassay method. All the pharmacological compounds used for the study were purchased from Sigma-Aldrich.

### 2.4. Statistical Analysis

Data are presented as mean ± standard error of the mean (S.E.M.). Student's* t*-test was performed using GraphPad Prism version 6.01 for Windows (GraphPad Software, La Jolla, CA, USA).

## 3. Results

**Figure 1:**
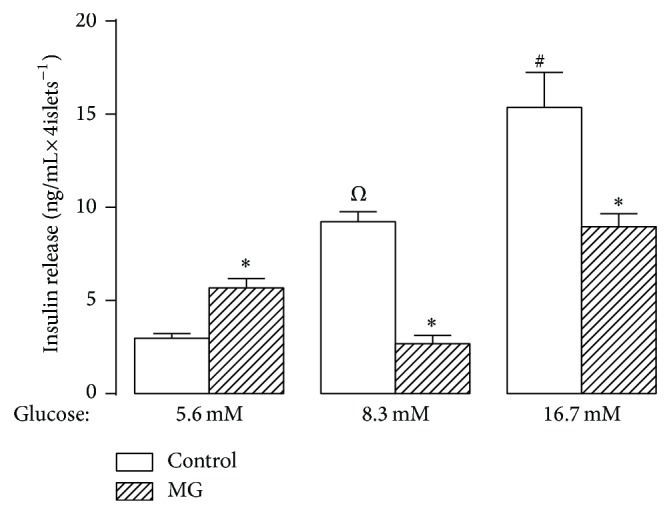
Glucose-stimulated insulin secretion (GSIS). Insulinotropic effect of GSIS from isolated pancreatic islets in absence or presence of methylglyoxal. Insulin secretion was stimulated by increasing concentrations of glucose, as indicated below the *x*-axis. Bars represent the mean ± S.E.M. ^∗^ *P* < 0.01 compared with the respective control group; ^*Ω*^ *P* < 0.01 compared with 5.6 or 16.7 mM of glucose; ^#^ *P* < 0.01 compared with 5.6 or 8.3 mM of glucose.

To study the effect of MG on insulin release triggered by different concentrations of glucose, cells were incubated with 5.6 mM, 8.3 mM, and 16.7 mM of glucose in presence or absence of MG (10 mM). As shown in Figure 1, at basal glucose concentration, MG induced an increase of 91% (*P* < 0.01) in the glucose-stimulated insulin secretion (GSIS). However, at 8.3 and 16.7 mM of glucose, the insulin secretion in presence of MG was reduced by 71% and 42% (*P* < 0.01), respectively.

**Figure 2:**
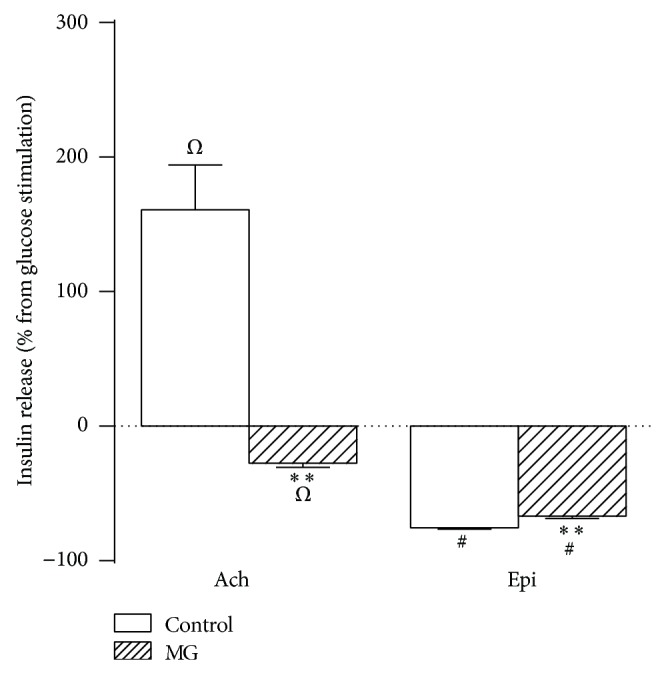
Effect of acetylcholine or epinephrine on GSIS. Insulin secretion was stimulated or inhibited by acetylcholine or epinephrine, as indicated below the *x*-axis, in absence or presence of methylglyoxal. Bars represent the mean ± S.E.M. of the percentage of insulin release compared with their respective glucose concentrations, which are represented by the line from 0. Ach, 10 *μ*M of acetylcholine in presence of 10 *μ*M of neostigmine + 8.3 mM of glucose; Epi, 1 *μ*M epinephrine + 16.7 mM of glucose. ^*Ω*^ *P* < 0.001 compared with 8.3 mM of glucose; ^#^ *P* < 0.001 compared with 16.7 mM of glucose; ^∗^ *P* < 0.05 and ^∗∗^ *P* < 0.001 compared with the respective control group.

**Figure 3:**
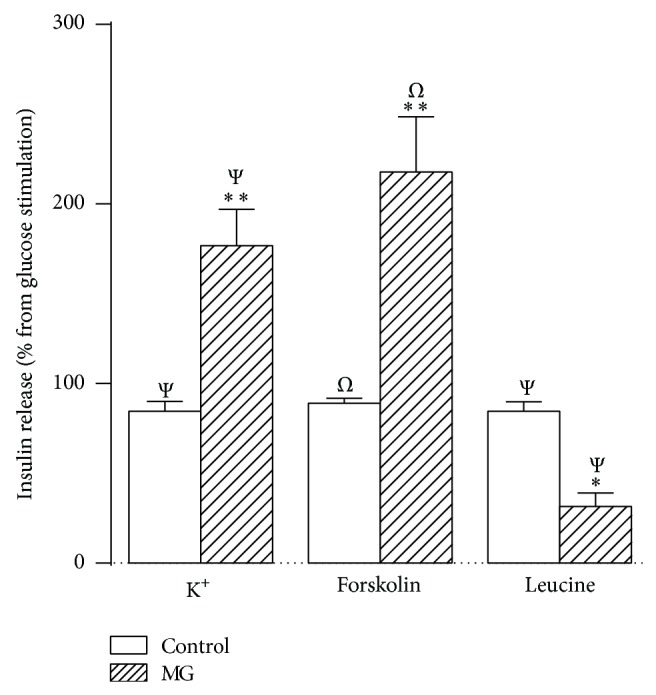
Effect of K^+^, forskolin, or leucine on GSIS. Insulin secretion was stimulated or inhibited by K^+^, forskolin, or leucine, as indicated below the *x*-axis, in absence or presence of methylglyoxal. Bars represent the mean ± S.E.M. of the percentage of insulin release compared with their respective glucose concentrations, which are represented by the line from 0. K^+^, 40 mM of potassium + 5.6 mM of glucose; forskolin, 10 *μ*M of forskolin + 8.3 mM of glucose; leucine, 10 mM of leucine + 5.6 mM of glucose. ^Ψ^ *P* < 0.001 compared with 5.6 mM of glucose; ^*Ω*^ *P* < 0.001 compared with 8.3 mM of glucose; ^#^ *P* < 0.001 compared with 16.7 mM of glucose; ^∗^ *P* < 0.05 and ^∗∗^ *P* < 0.001 compared with the respective control group.

Secondly, we studied the effect of MG on islets insulin secretion in presence of different secretagogues. Results shown in Figures 2 and 3 have been expressed as a percentage from the respective basal glucose concentration. In presence of 8.3 mM of glucose, acetylcholine amplified the insulin secretion by 160% (*P* < 0.001), and the presence of MG decreased the acetylcholine-increased insulin secretion by 27% (*P* < 0.001), compared with 8.3 mM of glucose. In presence of 16.7 mM of glucose, epinephrine decreased the insulin secretion by 76% (*P* < 0.001) and the addition of MG resulted in a slight decrease (67% compared with 16.7 mM of glucose, *P* < 0.001) of the epinephrine-induced inhibition of GSIS. In Figure 3, the GSIS was increased by 85% (*P* < 0.001) in presence of K^+^ and 5.6 mM of glucose, and the addition of MG induced by 177% (*P* < 0.001) the insulin secretion compared with 5.6 mM of glucose. Forskolin induced by 89% (*P* < 0.001) the insulin secretion in presence of 8.3 mM of glucose, while the addition of MG resulted in further increase of around 3-fold (*P* < 0.001). Finally, in presence of 5.6 mM of glucose, leucine increased the insulin secretion by 85% (*P* < 0.001), while in presence of MG this leucine-induced increase of insulin secretion was of 32% (*P* < 0.05) compared with 5.6 mM of glucose.

## 4. Discussion

Our study provides evidence to support the hypothesis that MG affects the insulin secretion in a dual manner. We have shown that short exposure of a low concentration of MG leads to an imbalance of insulin secretion at both basal and high glucose concentrations as well as in the presence of acetylcholine, epinephrine, potassium, leucine, and forskolin. These results indicate that different pathways are involved in AGEs regulation of insulin secretion and that their mode of action is dependent either on glucose concentration or on the nature of secretagogue applied in the cells. In agreement with a previous study [17], our findings support the idea of a dual effect of AGEs in the regulation of insulin secretion.

Glucose is the main nutrient that initiates the insulin secretion on pancreatic islets. It is internalized by glucose transporters 2 (GLUT2) present in the beta-cell surface. Briefly, within the cells, the glucose is metabolized by oxidative glycolysis, which leads to a rise of the ATP-to-ADP ratio. The increased levels of ATP result in the closure of ATP-sensitive potassium channels, which leads to a depolarization of the membrane and subsequent opening of voltage-dependent calcium channels and the influx of calcium resulting in the rise of cytoplasmic-free calcium concentration and activation of the exocytotic machinery [9]. The incubation of isolated islets with glucose stimulates the insulin secretion in a concentration-dependent manner [18]. Interestingly, when the islets were incubated with basal concentration of glucose and AGEs, the insulin release was increased. On the other hand, islets incubated with higher glucose concentrations and AGEs exhibited a great inhibition of the insulin release. These data indicate a possible interaction of AGE in a dual manner on the regulation of glucose-stimulated insulin secretion (GSIS). This AGE regulation could be related to the metabolic state of the cell. A previous study has shown that MG exerts a major damaging effect on INS-1E cells impairing both insulin action and secretion, which includes impairment of the insulin-induced phosphorylation of the insulin receptor (IRS) and activation of the glycogen synthase kinase 3 (GSK-3) with its reduced phosphorylation response to insulin [19].

To further investigate the AGE action on insulin secretion, we investigated the involvement of ATP-regulated potassium channels (K_ATP_^+^). The blocking of the K_ATP_^+^ by high extracellular potassium concentration led to increased insulin secretion even in low levels of glucose, as seen in the literature and in our results. Furthermore, the addition of AGEs induced even higher secretion in combination with extracellular potassium. An amplifying pathway augments insulin secretion without a reduced K_ATP_^+^ permeability (K_ATP_^+^ independent pathway), indicating that glucose controls the insulin secretion by other additional pathways [9, 20, 21]. This suggests that AGEs recruit the K_ATP_^+^ independent pathway.

Acetylcholine is one potent modulator of GSIS through its action on cholinergic muscarinic receptors present throughout the insulin secreting cells. When incubated with glucose and acetylcholine, the insulin release was highly increased. Otherwise, when these cells received also the AGE compound, they exhibited a rough decrease in insulin secretion, even lower than the secretion exhibited by islets only in presence of glucose. In agreement with previous studies, it seems that AGEs activate pathways of beta-cell damage, through generation of mitochondrial superoxide, which can lead to an impairment of insulin secretion [17, 22]. The cholinergic muscarinic receptors are classified as G protein coupled receptors (GPCR) and are regulated by phosphorylation following acetylcholine stimulation. These receptors couple with various downstream pathways such as the increase or decrease in cyclic AMP (cAMP) levels and stimulation of phospholipase C (PLC) with the formation of IP3 [23, 24]. In the same line, the adrenergic receptors are also formed of GPCR, but are targets of the catecholamines, and islets incubated with epinephrine presented a great inhibition of GSIS. On the other hand, the addition of AGEs caused a slight decrease in the epinephrine-induced inhibition of insulin release. To further explore the involvement of AGEs and cAMP levels, we activated the adenyl cyclase (AC) with forskolin [25], which resulted in increased insulin secretion when islets were incubated with only 8.3 mM of glucose. Adding the AGEs to this AC-activated pathway assay resulted in around 2-fold more insulin release. It is evident that AGEs may have a cross-talk interaction with the AC downstream pathway. Whether the AGEs are acting or not in those downstream pathways remains to be further studied.

We further analyzed the insulin secretion stimulated by leucine and MG. Leucine is known to stimulate insulin release in normal beta-cells by two mechanisms. Firstly, by degradation through transamination pathway producing *α*-ketoisocaproate, a metabolite which is a potent secretagogue allowing stimulation of insulin secretion through subsequent mitochondrial oxidation. Secondly, by the allosteric activation of glutamate dehydrogenase resulting in increased glutaminolysis, which also stimulates mitochondrial oxidation. Both mechanisms lead to an increase of the metabolic flux rate through the citric acid cycle, which increases the ATP production [26–28]. Our findings show that insulin release was increased in a basal concentration of glucose in presence of leucine; however, in the presence of AGEs this leucine-increased insulin secretion is blocked and is similar to the secretion levels from the control situation (5.6 mM of glucose). This latter result provides an insight that AGEs may be acting directly in the mitochondria, which is in agreement with a previous study supporting the idea that AGEs impair the secretion of pancreatic beta-cells at least in part through oxidative stress mechanisms [29]. Impaired mitochondrial function is also reported both* in vivo* and* in vitro* as a cause of insulin secretory dysfunction resulting from AGEs treatment [14, 15]. Another study has pointed out that the effects of AGEs, in a low concentration for longer exposure period, are mainly caused through a reduction in the expression of the malate dehydrogenase (*Mdh1/2*) gene, which plays an important role within the mitochondria [30]. Beyond the oxidative stress, AGEs are also formed in presence of hyperglycemia, but they still can be obtained from the diet [6–8, 31]. AGEs bind to their receptors for AGE (RAGE) resulting in a downstream signaling activation, generation of intracellular oxygen free radicals, and the activation of gene expression [22, 32] that could be involved in the inhibition of leucine-potentiated insulin release.

## 5. Conclusions

Altogether, we provide several evidences that AGEs might regulate the insulin secretion through specific and different pathways within the pancreatic islets, which remain to be revealed. Recent findings together with our data bring up the idea that AGEs can effectively inhibit or stimulate insulin secretion pathways. In addition to their chronically deleterious long-term effects in beta-cells, the AGEs acute effects described in the present work may participate in the onset of diabetes.
